# CABOCOL-01 trial: a single-arm phase II study assessing safety and efficacy of Cabozantinib for advanced or metastatic cervical carcinoma after platinum treatment failure

**DOI:** 10.1186/s12885-021-08758-9

**Published:** 2021-09-25

**Authors:** Elodie Coquan, Pierre-Emmanuel Brachet, Idlir Licaj, Alexandra Leconte, Marie Castera, Justine Lequesne, Emeline Meriaux, Isabelle Bonnet, Anais Lelaidier, Bénédicte Clarisse, Florence Joly

**Affiliations:** 1 Medical Oncology Department, Centre François Baclesse, F-14000 Caen, France; 2 Clinical Research Department, Centre François Baclesse, F-14000 Caen, France; 3 Data Processing Center of the North-West Canceropole (Centre de Traitement des Données du Cancéropôle Nord-Ouest), Centre François Baclesse, F-14000 Caen, France; 4 Normandy University, F-14000 Caen, France

**Keywords:** Cabozantinib, Metastatic cervical carcinoma, Quality of life, Anti angiogenic treatment

## Abstract

**Background:**

Cervical cancer is the tenth diagnosed cancer in the world. Early-stage and locally recurrent disease may be cured with radical surgery or chemo-radiotherapy. However, if disease persists or recurs, options are limited and the prognosis is poor. In addition to chemotherapy, bevacizumab, an antiangiogenic agent, has recently demonstrated its efficacy in this setting. Cabozantinib is an oral small molecule tyrosine kinase inhibitor that exhibits potent inhibitory activity against several receptor tyrosine kinases that are known to influence tumor growth, metastasis, and angiogenesis. The main targets of Cabozantinib are VEGFR2, MET and AXL. It is currently approved for the treatment of metastatic renal cell carcinoma, hepatocellular carcinoma and medullary thyroid carcinoma. Given its angiogenic properties associated with growth factor receptors inhibition, Cabozantinib represents a potential active treatment in cervical carcinoma. In this context, we propose to assess the efficacy and safety of cabozantinib monotherapy in advanced/metastatic cervical carcinoma (CC) after failure to platinum-based regimen treatment.

**Methods:**

This study is a single-arm two-stage multicenter phase II aiming to simultaneously assess efficacy and safety of Cabozantinib among advanced/metastatic cervical carcinoma (CC) after failure to platinum-based regimen treatment. The main criterion will be based on both safety and clinical efficacy by conducting a Bryant-and-Day design. Safety endpoint is the proportion of patients with clinical gastro-intestinal (GI) perforation/fistula, GI-vaginal fistula and genito-urinary (GU) fistula events grade ≥ 2 (NCI CTCAE V.5.0) occurring up to one month after the end of treatment. Efficacy endpoint is the proportion of patients with disease control rate 3 months after Cabozantinib initiation. A patients’ self-reported quality of life evaluation is also planned, as well as the investigation of nutritional outcomes. Cabozantinib will be administered at the daily dose of 60 mg given orally, without interruption until disease progression or discontinuation for any cause.

**Discussion:**

Cabozantinib is a promising drug for patients with advanced/metastatic cervical cancer where few therapeutics options are available after failure to platinum-based regimen metastatic CC. It appears challenging to assess the interest of Cabozantinib in this indication, taking into account the potential toxicity of the drug.

**Trial registration:**

NCT04205799, registered “2019 12 19”.

**Protocol version:**

Version 3.1 dated from 2020 08 31.

## Background

### Cervical carcinoma management

Cervical carcinoma (CC) is the tenth diagnosed cancer and leading cause of cancer death in the world [1]. The most significant cause of CC is persistent human papillomavirus infection (HPV) detected in 99% of cervical tumors. Early-stage and locally recurrent disease may be cured with radical surgery, chemo-radiotherapy or both [2]. However, if disease persists or recurs, options are limited and the prognosis is poor.

Palliative chemotherapy with cisplatin 50 mg/m^2^ every 3 weeks was the standard of care of advanced/metastatic CC, until cisplatin-based doublets with topotecan or paclitaxel demonstrated their superiority in terms of response rate (RR) and progression-free survival (PFS): objective responses occurred in 36% of patients receiving cisplatin and paclitaxel (versus 19% for cisplatin alone) [3, 4]. The median PFS was 4.8 months for the combination but there was no difference in median overall survival (OS) (9.7 months).

Vascular endothelial growth factor (VEGF) promotes angiogenesis and is an important mediator of disease progression in CC. The GOG-240 study explored the addition of bevacizumab, an antibody against VEGF, to chemotherapy in a randomized phase III trial in primary stage IVb or recurrent/persistent disease [5]: patients were randomized to paclitaxel-cisplatin or paclitaxel-topotecan, both with or without bevacizumab. With the combined data for the two chemotherapy regimens, the addition of bevacizumab to chemotherapy was associated with increased OS (17.0 months vs. 13.3 months, *P* = 0.004) and higher RR (48% vs. 36%, *P* = 0.008). Importantly, with bevacizumab treatment, or more generally with antiangiogenic treatment, increased reports of fistulas have been reported in previous studies in CC. In the GOG-240 study, 32 (15%) of 220 patients in the chemotherapy plus bevacizumab group had fistulas, compared with 3 (1%) in the chemotherapy-alone group. In both groups, patients who developed fistula were previously treated with pelvic radiotherapy [6]. Thirteen (6%) patients had clinically significant or severe (ie, grade 3) fistula in the chemotherapy plus bevacizumab group versus one (< 1%) in the chemotherapy-alone group. No fistula resulted in surgical emergencies, sepsis, or death. In addition to pelvic irradiation, other factors were associated with fistulas, including pelvic recurrence, pre-existing hypertension, and current tobacco use. In their real life data study, Godoy-Ortiz et al. reported increased rate of fistula (22%) over 27 patients treated with bevacizumab [7]. Palavalli studied predictive factors of fistula event in 74 patients treated with bevacizumab for advanced, recurrent or metastatic CC [8]. Lower albumin levels and use of bevacizumab were identified as independent predictor factors for fistula onset (*P* = 0.004 and *P* = 0.024, respectively).

Despite the increased toxicity rate, there was no deterioration in health-related quality of life [9] in bevacizumab arm in the GOG 240 trial. So, bevacizumab associated with a doublet of platinum chemotherapy become the standard of care for the first line regimen in metastatic or recurrent CC which is not eligible to local treatment [2].

Other different additional agents that target VEGF have been investigated in advanced CC.

Thus, Monk et al. enrolled a total of 410 patients to assess the efficacy of Pazopanib, Lapatinib or the combination of both, in women with metastatic, persistent or recurrent cervical cancer [10]. Unfortunately, the study prematurely discontinued for futility and excessive toxicity of the combination, although patients who received pazopanib in monotherapy experienced a higher PFS and longer median OS (12.4 months) when compared with patients treated with lapatinib. Diarrhea was the most common grade 3 adverse event (11%). Because only 50% of the total expected patients participated in the final analysis, it was not possible to draw any definitive conclusions regarding the real effectiveness of the therapy.

In a phase II study, Sunitinib was administered to 19 patients with unresectable, locally advanced or metastatic cervical cancer [11]. Sixteen patients (84%) had a stable disease as their best response with a median duration of 4.4 months. Five patients (26.3%), who had previously received either chemo-radiation or radiation therapy, developed a fistula during or after sunitinib assumption. It was therefore concluded that the drug has insufficient activity and was too toxic in this clinical setting.

A randomized phase II study evaluated the efficacy of Cediranib associated with carboplatin and paclitaxel in the first line treatment of metastatic/advanced cervical cancer [12]. The trial closed prematurely owing to withdrawal of drug supply but results showed a significant efficacy of the combination (median PFS 8.1 months versus 6.7 months in the placebo group, hazard ratio 0.58, *P* = 0.032). This finding was accompanied by an increase in toxic effects, mainly diarrhea, hypertension, and febrile neutropenia.

Lastly, Brivanib, an oral, tyrosine kinase inhibitor against VEGF and fibroblast growth factor receptor was investigated in a phase II study. The median PFS was 3.2 months (90% CI: 2.1–4.4) and the median OS was 7.9 months (90% CI: 6.1–11.7), with a manageable toxicity profile but trial was stopped due to lack of drug availability [13].

Following progression after first-line chemotherapy, different cytostatic agents such as docetaxel, topotecan or gemcitabine, have been evaluated [14–16]. However, response rates are low and duration of response is short.

Very recently, pembrolizumab was investigated in 98 patients with recurrent or metastatic cervical cancer who had received at least one line of chemotherapy for metastatic disease in a cohort of Keynote 158, a multicenter, non-randomized, open-label, multi-cohort trial [17]. Patients were treated with Pembrolizumab until unacceptable toxicity or documented disease progression. Among the 98 patients, 77 (79%) patients had tumors that expressed PD-L1 with a combined positive score (CPS) ≥1. With a median follow-up time of 11.7 months, the overall response rate (ORR) in CPS positive tumors was 14.3% (95% CI: 7.4, 24.1), including 2.6% complete responses and 11.7% partial responses. Based on these results, the Food and Drug Administration approved pembrolizumab, in June 2018, for recurrent or metastatic cervical cancer patients with disease progression or after chemotherapy, whose tumor expresses PD-L1 (CPS ≥1).

However, in Europe, to date, no standard second-line chemotherapy is recommended in guidelines [2].

### Cabozantinib drug

Cabozantinib is an oral small molecule tyrosine kinase inhibitor that exhibits potent inhibitory activity against several receptor tyrosine kinases that are known to influence tumor growth, metastasis, and angiogenesis [18]. The main targets of cabozantinib are VEGFR2, MET, RET and AXL. In Phase I and Phase II studies, Cabozantinib has demonstrated antitumor activity in multiple tumor types. It is currently approved for the treatment of metastatic renal cell carcinoma, hepatocellular carcinoma and medullary thyroid carcinoma. The most common grade 3 or 4 adverse events of Cabozantinib in clinical trials are hypertension, diarrhea, fatigue, palmar-plantar erythrodysesthesia syndrome and anaemia which are common side effects of antiangiogenic treatments.

Beyond its angiogenic properties, Cabozantinib represents a potential active treatment in CC taking into account its inhibition over the growth factor receptors cMET and AXL.

In CABOSUN and in METEOR trials evaluating Cabozantinib in metastatic renal cell carcinoma, MET surexpression assessed by immunochemistry showed a trend toward more benefit in MET-positive tumors but it was not statistically significant, even with combined data of both trials [19–21].

Development of CC is a multistep process initiated by persistent infection with high-risk HPV. The most important prognostic variables of cervical carcinoma are represented by clinical or histological data (International Federation of Gynecology and Obstetrics FIGO stage, lymph-vascular space involvement). Recently, there has been an increasing interest in the identification of biomarkers able to predict both response to treatment and survival. c-MET receptor and its ligand, the hepatocyte growth factor (HGF) is known to play an important role in cancer growth and metastasis as well as development of drug resistance, especially with VEGFR inhibitors [22]. In cervical cancer, some authors report that c-MET and HGF overexpression tend to have a prognostic value. Zhang and al found that patients with squamous cell CC had an elevated serum HGF compared to patients with cervical intraepithelial neoplasia or healthy controls [23]. Furthermore, HGF serum concentrations were related to the pathological grade and the clinical, FIGO stage. Interestingly, the serum levels of HGF were also reported to be higher in patients with metastasis compared to those without metastasis. HGF expression may be correlated with cancer cell metastasis and infiltration but the latter did not reach statistical significance. Refaat et al. assessed retrospectively the association between pre-treatment c-MET expression and treatment outcome in 28 patients treated for an advanced CC with concurrent chemo-radiation therapy and showed an association between c-MET overexpression and poor OS, PFS, distant metastasis control and loco-regional control [24]. A meta-analysis showed that c-Met expression was higher in CC (61.0%) than in non-neoplastic cervical tissue (19.7%) and confirmed the correlation between high level c-MET expression and disease-free survival, lymph node involvement, and lymphovascular invasion [25]. However, the molecular mechanisms underlying the upregulation of this proto-oncogene are unknown. Chen and al found a relationship between the oncogenic E6 proteins expressed by high-risk HPV isotypes and epigenetic activation of super-enhancers in the genome that drive expression of key oncogenes like epidermal growth factor receptor and c-MET [26]. A study showed that downregulation of MET receptor expression via RNA interference in different CC cell lines dramatically decreased tumor growth and forced tumor differentiation in vivo [27]. MET receptor silencing also led to a dramatic decrease in cell size and a decrease in proliferation rate under normal and stress conditions. These findings highlight the role of the MET receptor in CC cells and indicate the MET receptor as a potential therapeutic target for advanced CC.

Axl receptor tyrosine kinase is involved in the tumorigenesis and metastasis of many cancers but its role in HPV induced tumors is not completely established. In a recent report, authors showed that AXL expression was higher in HPV type 16E6 (HPV16E6)-overexpressing HeLa cells than in the controls and the expression of AXL was correlated with clinical stage of cervical cancer and HPV16/18 infection [28]. AXL expression was induced in HPV16E6 cervical cancer cells, suggesting that blockade of AXL signaling might be an effective way to reduce the progression of cervical cancer.

### Purpose

Taking into account its several targets inhibition, Cabozantinib seems to be an interesting drug for advanced/metastatic cervical cancer where few therapeutics options are available after failure to platinum-based regimen. However, given the previous findings concerning the safety profile of other antiangiogenic drugs in this setting, a specific attention had to be paid on toxicities under Cabozantinib treatment. In this context, we propose to implement a phase II trial aiming to simultaneously assess efficacy and safety of Cabozantinib monotherapy in advanced/metastatic cervical carcinoma after failure to platinum-based regimen treatment.

## Methods / design

We propose a single-arm two-stage multicenter phase II study aiming to simultaneously assess efficacy and safety of Cabozantinib. The CABOCOL-01 protocol and this manuscript have been written in accordance with standard protocol items, namely recommendations for interventional trials (SPIRIT).

### Primary outcome

The main objective of the study is based on joint primary endpoints of efficacy and safety as proposed by the Bryant-and-Day design [29]:

- **Efficacy** will be assessed by the proportion of patients with disease control rate 3 months after Cabozantinib treatment initiation.
- **Safety** will be assessed by the proportion of patients with clinical gastro-intestinal (GI) perforation/fistula, GI-vaginal fistula and genito-urinary (GU) fistula events grade ≥ 2 from National Cancer Institute Common Terminology Criteria for Adverse Events (NCI CTCAE), version 5.0 criteria.

### Secondary outcomes

The secondary objectives are to evaluate:

- The PFS time, defined as the time between initiation of Cabozantinib treatment and progression (RECIST 1.1 criteria) and death of any cause whichever occurs first.
- The ORR defined as the percentage of patients who have achieved complete response or partial response with RECIST 1.1 criteria.
- The OS time, defined as the time between initiation of Cabozantinib treatment and death of whatever cause.
- Toxicities evaluated according to NCI CTCAE version 5.0 criteria, in terms of kind, grade, time of onset, reversibility
- Significant toxicities responsible for interruption or dose reduction of Cabozantinib treatment
- Proportion of patients requiring Cabozantinib dose reduction or treatment stopping for toxicity
- Scores of Quality-of-life (QoL) according to French versions of the self-administered standardized validated questionnaire: EORTC QLQ-C30 and its additional cervix cancer module (QLQ–CX24)
- To investigate nutritional outcomes, including weight, sarcopenia, biological markers, muscle mass, and muscle strength under Cabozantinib in advanced CC

### Explorative outcomes

We will constitute a biological collection (blood, tumoral tissue) for further biological explorations such as the evaluation of (i) the impact of angiogenesis biomarkers and intra-cellular activated pathways in terms of oncological outcomes and response to Cabozantinib, and (ii) the predictive value of HPV circulating tumoral DNA during Cabozantinib treatment.

### Study population

Eligibility criteria are precised in Table 1. The CABOCOL-01 study addresses patients with recurrent unresectable or metastatic cervix carcinoma with squamous cell, adenocarcinoma or adenosquamous histology after at least on prior platinum-based chemotherapy for metastatic/recurrent disease.

**Table 1:** Study eligibility criteria

Inclusion criteria	- Female 18 years of age or older, with histologically confirmed recurrent unresectable or metastatic cervix carcinoma with squamous cell, adenocarcinoma or adenosquamous histology.
	- Patient may have received at least one prior chemotherapy regimen of platinum-based chemotherapy for recurrence or metastatic disease. Cisplatin given in combination with radiation for a localized disease does not count as a prior chemotherapy. Prior treatment for advanced/metastatic disease with bevacizumab and/or immune checkpoint inhibitors are allowed. - ECOG performance status 0–2
	- Measurable disease per RECIST 1.1
	- The subject must have recovered to baseline or CTCAE version .5.0 ≤ Grade 1 from clinical toxicities related to any prior treatments, i.e. chemotherapy or pelvis radiation unless AE(s) are clinically non-significant (for example alopecia)
	- Adequate hematological, renal (Calculated creatinine clearance ≥30 mL/min by the CKD-EPI method) and hepatic function.
	- Serum albumin ≥3.0 g/dL (≥ 30 g/L)
	- Left-ventricular ejection fraction ≥50%
	- Subjects affiliated to the social security system
Exclusion criteria	- Clinically significant gastrointestinal abnormalities that may affect absorption of cabozantinib including, but not limited to: malabsorption syndrome, major resection of the stomach or small bowel.
	- Clinically significant gastrointestinal abnormalities that may increase the risk for gastrointestinal bleeding and/or fistula, history of abdominal fistula, perforation or intra-abdominal abscess, gastro-intestinal obstruction
	- History of any one or more of the following cardiovascular conditions within the past 6 months: cardiac angioplasty or stenting, myocardial infarction, unstable angina, coronary artery bypass surgery, symptomatic peripheral vascular disease, class III or IV congestive heart failure, as defined by the New York Heart Association.
	- History of cerebrovascular accident including transient ischemic attack within the past 6 months. Subjects with recent DVT or asymptomatic pulmonary embolism who have been treated with therapeutic anti-coagulating agents for at least 4 weeks are eligible.
	- Corrected QT interval (QTc) > 500 msec.
	- Uncontrolled hypertension defined as systolic blood pressure of > 150 mmHg or diastolic blood pressure of > 100 mmHg despite an optimal treatment.
	- Major surgery or trauma within 28 days prior to first dose of investigational product and/or presence of any non-healing wound, fracture, or ulcer.
	- Evidence of active bleeding or pathologic conditions that carry high risk of bleeding such as known bleeding disorders, coagulopathy or tumor involving major vessels.
	- Presence of brain metastases or epidural disease unless adequately treated with radiotherapy and/or surgery (including radiosurgery) and stable for at least 3 months before inclusion. Eligible subjects must be neurologically asymptomatic and without corticosteroid treatment at the time of inclusion.
	- Concomitant use of known strong cytochrome 3A4 inhibitors or inducers.
	- Patients with second primary cancer, except adequately treated non-melanoma skin cancer, or other solid tumors curatively treated with no evidence of disease for ≥3 years

### Study sites

The list of study sites is indicated on https://clinicaltrials.gov/ct2/show/NCT04205799. The participation of 8 French Comprehensive Cancer Centres highly involved in the ARCAGY/GINECO intergroup is planned (Table 2).

**Table 2:** Participating centers

INVESTIGATORS	PARTICIPATING FRENCH COMPREHENSIVE CANCER CENTRES
**Investigateur principal:** Dr. Elodie COQUAN **Co-investigateurs:** Pr Florence JOLY Dr. Emeline MERIAUX Dr. Pierre-Emmanuel BRACHET Dr. Mélanie DOS SANTOS Dr. Georges EMILE Dr. Isabelle BONNET Dr. Alison JOHNSON	**Centre François Baclesse, CAEN**
**Investigateur principal:** Pr Isabelle RAY-COQUARD **Co-investigateurs:** Dr. Olivier TREDAN Dr. Lauriane EBERST Dr. Philippe TOUSSAINT	**Centre Léon Bérard, LYON**
**Investigateur principal:** Dr. Jean-Sébastien FRENEL **Co-investigateurs:** Dr. Dominique BERTON Dr. Ludovic DOUCET Dr. Emmanuelle BOURBOULOUX Dr. Carole GOURMELON Dr. Pauline DU RUSQUEC Dr. Audrey ROLLOT Dr. Judith RAIMBOURG **Investigateur principal:** Dr. Sophie ABASIE LACOURTOISIE **Co-investigateurs:** Dr. Frédéric BIGOT Dr. Victor SIMMET Dr. Patrick SOULIE Dr. Anne PATSOURIS Dr. Paule AUGEREAU Dr. Elouen BOUGHALEM Dr. Margot NOBLECOURT	**Institut de Cancérologie de l’Ouest, site NANTES** **Institut de Cancérologie de l’Ouest, site ANGERS**
**Investigateur principal:** Dr. Coraline DUBOT **Co-investigateurs** Dr. Manuel RODRIGUES Dr. Sophie FRANCK Dr. Anne DONNADIEU Dr. Diana BELLO-ROUFAI Dr. Patricia TRESCA Pr Roman ROUZIER Dr. Eugénie GUILLOT Dr. Delphine HEQUET Dr. Claire BONNEAU	**Institut CURIE, PARIS**
**Investigateur principal:** Dr. Cyril ABDEDDAIM **Co-investigateurs:** Dr. Annick CHEVALIER-PLACE Dr. Valérie CHEVALIER EVAIN	**Centre Oscar LAMBRET, LILLE**
**Investigateur principal:** Dr. Fanny POMMERET **Co-investigateurs:** Dr. Patricia PAUTIER Dr. Emeline COLOMBA-BLAMEBLE Dr. Alexandra LEARY	**Gustave Roussy, VILLEJUIF**
**Investigateur principal:** Pr Véronique D’HONDT **Co-investigateurs:** Dr. Michel FABBRO	**Institut régional du Cancer, MONTPELLIER**

### Study treatments and procedures

**Fig. 1:**
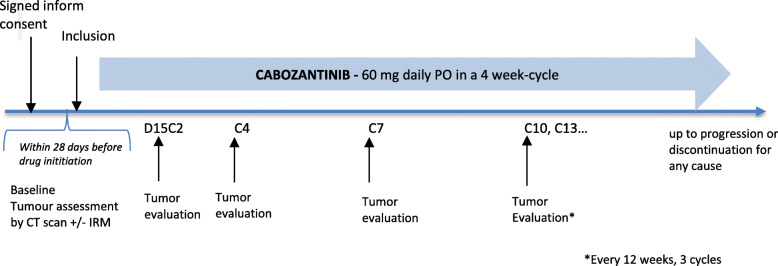
CABOCOL-01 study schedule

The study schedule is resumed in Fig. 1 and an overview of study assessments and procedures is presented in Table 3.

**Table 3:** CABOCOL-01 study procedures

	Before inclusion within 28 days before drug initiation	During treatment (1 cycle = 28 days)						End of treatment 30 days after the last dose of study treatment (+/− 7 days)	Follow-up every 3 months up to progression	Overall survival after disease progression
		Cycle 1		Cycle 2		Other Cycles from cycle 2	Additional Assessment at D1C4 and every 3 cycles (D1C7, D1C10 …) (within 7 days)			
		D1	D15	D1	D15	D1				
**Study drug administration**		CABOZANTINIB treatment (in a 28 day-cycle)								
**Informed Consent** *should be done before any study procedures*	**✓**									No study visit is required. The following treatment is at the discretion of physician
**Clinical assessment** - Complete physical examination including gynecological examination, weight, height (only at baseline), ECOG, vital signs - Adverse Events collection and concomitant treatments	**✓**	**✓**	**✓** **✓**	**✓** **✓**	**✓** **✓**	**✓** **✓**		**✓** **✓**	**✓** **✓**	
**Laboratory Assessments**^**1**^ - Blood assessment - Urinary assessment	**✓** within 14 days before drug initiation	**✓**^**3**^	**✓** within 3 days	**✓** within 3 days	**✓** within 3 days	**✓** within 3 days		**✓** within 3 days	**✓** within 3 days	
**Cardiac assessment** - ECG (QT interval) - Cardiac Echography or MUGA	**✓** **✓**			**✓**			**✓**			
**Quality-of-life assessment** EORTC QLQ-C30 / CX24	**✓**				**✓**		**✓**			
**Diary card**		**✓**^**4**^		**✓**		**✓**				
**Radiological assessment** - CT-scan (thorax, abdominal and pelvis) ^6^ - Pelvic MRI ^**2**^	**✓** **✓**				within 3 days **✓** **✓**		**+/− 7 days** **✓** **✓**		**+/− 14 days** **✓** **✓**	
**Biological collections** - Tumoral biopsy optional^5^ - Blood samples	**✓** **✓**			**✓**	**✓**		**✓at progression**			

^*1*^*Laboratory assessment*

• Hematology *(CBC, platelets)*

• Serum biochemistry (Albumin, total alkaline phosphatase (ALP), alanine amino transferase (ALT), aspartate amino transferase (AST), corrected calcium, creatinine and clearance by CKD-EPI method, glutamyltransferase (GGT), glucose, lactate deshydrogenase (LDH), magnesium, phosphorus, potassium, sodium, total bilirubin (conjugated and unconjugated if clinically indicated), total protein

• Coagulation (PT/INR and PTT) mandatory before inclusion, then for other visits only if clinically indicated

• Thyroid function tests (TSH, free T3, free T4*) (at each biological assessment except D15C1 and D15C2)*

• Tumor marker: SCC antigen *(at each biological assessment except D15C1)*

• Urine analysis:

- Before inclusion: Urine analysis: urine protein-to-creatinine ratio (UPCR) ≤ 1 g/g (≤ 113.2 mg/mmol) creatinine or 24-h urine protein < 1 g. When a UPCR exceeds 1 g/g, a repeat UPCR or a 24-h urine protein and creatinine should be performed to confirm the result

-For other visits: Urine dipstick (with protein, blood and leucokytes).

*If results > 2+, an urinary analysis must be performed with a Urine protein / creatinine ratio (UPCR). When a UPCR exceeds 1 g/g, a repeat UPCR or a 24-h urine protein and creatinine should be performed to confirm the result.*

• Urinal pregnancy test *(*Women of childbearing potential), only before inclusion

^*2*^*Mandatory pelvis IRM at inclusion and optional for further evaluations. MRI can be used in addition of CT scan if a local recurrence could not be assessed by CT*

^*3*^*Only if realized more 14 days before D1*

^*4*^*Subjects should be instructed before the beginning of the treatment of the risk of diarrhea and the initial management. A preventive prescription can be given to the patient at the beginning of the treatment.*

^*5*^*Fresh biopsy from primitive and/or from metastatic sites if feasible and only if the patient agrees on the consent form. AND Mandatory: One paraffin block of archival initial or recurrent tumour will be sent to sponsor during study.*

^*6*^*A central review of the scanners will be done in the study to assess sarcopenia. An anonymized copy of the scanner imagery will be sent to the sponsor during the study*

Cabozantinib will be administered at the daily dose of 60 mg given orally in a 4-week cycle. Cabozantinib will be continued without interruption until disease progression or discontinuation for any cause. No premedication is required before Cabozantinib administration. In case of toxicity, a maximum of two dose reductions are allowed: it is recommended to first reduce to 40 mg daily, and then to 20 mg daily.

Because there is a potential for interaction of Cabozantinib with other concomitantly administered drugs through the cytochrome P450 system, the concurrent use of all other drugs, over-the-counter medications, or alternative therapies will be collected. Importantly, anticancer therapy, therapeutic doses of anticoagulants will not be allowed. As for palliative external radiation while on study, chronic co-administration of strong inducers or inhibitors of the CYP3A4 family, and co-administration with drugs associated with QTc prolongation, they should be avoided while on Cabozantinib treatment.

Tolerance assessment will be performed every 14 days during the first 2 months, and then every month by clinical examination and biological exams.

As part of this research, any fistula or gastro-intestinal perforation are considered as adverse events of interest.

Efficacy assessment will be performed at 6 weeks, 12 weeks and then every 12 weeks with CT scan. Pelvis MRI will be realised during the screening period and could be repeated as a systematic exam in addition to CT scan if a local recurrence could not be assessed by CT scan.

Quality-of-life will be measure using the validated self-questionnaires EORTC QLQ-C30 and QLQ-CX24 at 6 weeks, 12 weeks and then every 12 weeks (Table 2).

### Statistical design overview

CABOCOL-01 trial is a multicenter non-randomised phase II study.

#### Sample size calculation

We plan to use a single arm two-stage multicenter phase II trial based on a Bryant-and-Day design, selected in order to simultaneously assess efficacy and safety and to minimize the expected number of patients treated in case of insufficient efficacy and/or safety of cabozantinib monotherapy.

In accordance with literature estimating a median PFS around 3.4 months for advanced cervical cancer patients under bevacizumab monotherapy and 4 months for pazopanib, the null hypothesis for efficacy is a 3-month disease control rate of 30% and we expect a rate of 50% to conclude to efficacy of cabozantinib [11, 30].

Toxicity, defined as clinical significant (grade ≥ 2 NCI CTCAE version 5) fistula and perforation rate, will be considered as acceptable if it concerns at most 10% of patients and intolerable if it exceeds 25%.

According to these hypotheses, considering an alpha risk of 5% for efficacy and 10% for toxicity and a power of 80%, assuming a 10% drop-out rate, 57 patients are required in this study (25 for the first stage and 32 additional for the second stage).

Especially, 22 assessable patients will be included in the first stage. If disease control is observed in less than 8 patients at 3 months or if more than 4 patients experiment fistula or perforation, the study will be stopped at the interim analysis to conclude to insufficient efficacy or unacceptable toxicity of Cabozantinib. Eventually, observation of a minimum of 21 patients with disease control with less than 9 patients with toxicity will allow to conclude to efficacy and tolerance.

To be noted, enrolment were not initially planned to be halted to conduct the interim analysis. However, due to the quickness of recruitment in the study, the protocol was amended to suspend the inclusions to perform the interim analysis, in order to have a sufficient perspective on the tolerance and efficacy of Cabozantinib single-agent treatment.

#### Statistical analyses

Patients will be assessable for the efficacy analysis if they have a reported progression ≤3 months or a minimum follow-up of 3 months. Patients who drop out from the study prior to the 3 months will be included as failed treatment in the intent-to-treat analysis, but not included in the per-protocol analysis of efficacy.

Patients will be included in the safety analysis if they received at least one dose of Cabozantinib. Patients who were removed from the study due to adverse events will be followed-up until recovery or stabilization of symptoms.

Efficacy and safety will be evaluated simultaneously as part of the main objective.

The primary endpoints, the response rate and toxicity rate, will be evaluated at 3 months with their corresponding two-sided 95% CI.

For time-to-event endpoints, medians (if reached) will be presented and/or event rates at selected time points using the using the Kaplan-Meier method.

Frequency tables will be tabulated (overall and by tumor group) for all categorical variables and proportions will be estimated using the efficacy population as denominator (unless specifically specified otherwise). All estimates will be complemented with an appropriate 95% confidence interval where applicable. Efficacy and safety will be evaluated according to pre-treatment with Cabozantinib.

Adverse events and laboratory abnormalities observed during the study will be tabulated (worst CTC grade per patient) overall and further tabulation will be made based on time of occurrence and relationship to treatment. The latter excludes events unrelated or not likely related to treatment, but includes events for which the relationship with treatment is not assessable. Tolerance will be summarized by duration of treatment, reasons of discontinuation, dose reduction rates and reasons for dose reductions.

In addition, sensitivity analyses will be conducted among patients pretreated with bevacizumab. Following a A’Hern design in this subgroup, observation of a minimum of 8 disease control among 19 assessable bevacizumab pre-treated patients will be required to accept a 3-month control rate ≥ 50% against a rate of ≤20% with 80% power and a 2.5% significance level [31]. If the limit of 19 patients is not reached, efficacy and safety of the bevacizumab subgroup will be described in detail and we will further compute the posterior statistical power with the included patients.

### Patient reported outcomes

QoL scores and changes from baseline scores will be described for selected primary scales. Missing values will be considered such that if at least half the items from the scale will be completed, it will be assumed that the missing items will have values equal to the average of those items present. The Z-test or the non-parametric Wilcoxon–Mann–Whitney tests will be used to evaluate the evolution in global health status and other dimensions of the EORTC QLQ-C30 and subscale QLQ–CX24. In addition, an evaluation of the time to ≥10-point deterioration will be also carried out using survival analysis methods. A composite definition for deterioration based on death and tumor progression will be used. To account for missing data as a potential major source of bias, the compliance mechanism will be investigated prior to initiating the QoL analysis. Characteristics of patients with and without valid QoL data will be compared and trends over time per dropout pattern will be investigated. Center-stratified proportional hazard Cox regression model will be used in order to investigate whether the compliance mechanism is linked to selected prognostic variables.

### Data monitoring committee

An Independent Data Monitoring Committee (IDMC) will be set-up to ensure the protection of patients, to ensure the ethical conduct of the study, to evaluate the benefit/risk ratio of the study and to insure an independent review of the scientific outcomes during and at completion of the study.

The committee will include a biostatistician, a pharmacologist and a medical oncologist.

The members of the IDMC will be consulted before the trial initiation, after the enrolment of 10 patients, notably to pay a special attention on every AE of interest (fistula/perforation), thereafter, at the interim analysis and te final analysis.

### Data management

A Web Based Data Capture (WBDC) system will be used for data collection and query handling. The investigator will ensure that data are recorded on the eCRFs as specified in the study protocol and in accordance with the instructions provided.

The investigator ensures the accuracy, completeness, and timeliness of the data recorded and of the provision of answers to data queries according to the Clinical Study Agreement. The investigator will sign the completed eCRFs. A copy of the completed eCRFs will be archived at the study site.

### Withdrawal from study

The reasons for why a patient may discontinue to participate to the study or interrupt Cabozantinib treatment include the following circumstances:

- Disease progression
- Need to initiate another anti-tumor treatment (e.g., systemic anticancer treatment palliative radiation and surgery)
- Unacceptable toxicity, not compatible with study treatment
- Necessity for withholding study drug for greater than 4 weeks for study-treatment related AEs.
- Significant noncompliance with the protocol schedule in the opinion of the investigator or the Sponsor.
- Patient’s decision (the data already collected during the search can be kept and exploited unless the patient opposes it)
- Intercurrent illness or other reason that requires stopping treatment of the study
- Patient lost to view
- Investigator’s decision

### Ancillary studies

#### Tumoral circulating DNA

Circulating tumor DNA has been widely evaluated as a liquid biopsy for detecting cancer, monitoring disease, characterizing drug targets and uncovering resistance in various tumors. Viral DNA has been detected in the serum of patients with virally induced tumors. HPVs are small, non-enveloped viruses that induce squamous epithelial tumours like cervical carcinoma. Circulating cell free HPV DNA may serve as a unique tumor marker for HPV-associated malignancies [32]. Droplet-digital PCR is able to detect and quantify tumor-derived HPV DNA sequences in patient blood with high sensitivity and specificity [33, 34]. Here, circulating tumoral DNA will be collected at screening, at 1 month, then at 6 weeks and at progressive disease, corresponding to tumoral evaluation, to evaluate the usefulness of serum HPV DNA level as a marker for efficacy and early biomarkers of failure for Cabozantinib in CC.

Fresh biopsy will be obtained at baseline from primitive and/or from metastatic sites if feasible and if the patient agrees on the consent form and archival initial or recurrent tumor sample will also be used. Blood samples will be collected during the study. They will be used for searching biomarkers of efficacy to Cabozantinib treatment, especially c-MET expression.

#### Sarcopenia

Sarcopenia is a syndrome characterized by low skeletal muscle mass with impaired muscle function, with multifactorial etiology and it is a real concerns in metastatic CC [35]. Described with antiangiogenics, sarcopenia is related to higher toxicity and/or poor responses to antineoplastic drugs, and decreased survival in cancer patients [36]. Recent data showed sarcopenia is a frequent but unexplored adverse event of Cabozantinib [37]. We will investigate prospectively the impact of Cabozantinib on weight loss and sarcopenia in an homogenous population with high risk of malnutrition. Skelettal muscle index will be calculated and compared with a pre-specified sex-based threshold.

## Discussion

Angiogenesis is a well-known important target in metastatic CC. Bevacizumab has already demonstrated its efficacy and is used in this indication. Cabozantinib is a promising drug for CC thanks to its VEGF inhibition and other targets which can be involved in CC tumorogenesis and angiogenesis resistance mechanisms. Specially, in France, bevacizumab is not reimboursed in CC, which limits patient access to antiangiogenic treatment. The CABOCOL-01 trial will therefore access efficacy of Cabozantinib single-agent treatment in patients pretreated or not with becizumab leaded to have an overview of Cabozantinib efficacy in CC tumors naive or resistant to an anti-angiogenesis treatment. This study is challenging due to the potentiel toxicity of Cabozantinib in this population. The Bryant-and-Day two-stage phase II design was therefore specifically selected in order to assess the interest of Cabozantinib in this setting while considering both its efficacy and safety profile. Additionally, the CABOCOL-01 trial will be an oportunity to contribute to identify biomarkers of efficacy of Cabozantinib in CC.

## Data Availability

Not applicable.

## Abbreviations

CC: Cervical Carcinoma
CPS: Combined Positive Score
CT: Computed Tomography
CTCAE: Common Terminology Criteria for Adverse Events
FIGO: International Federation of Gynecology and Obstetrics
GI: Gastro-Intestinal
GU: Genito-Urinary
HGF: Hepatocyte Growth Factor
HPV: Human Papilloma Virus
IDMC: Independent Data Monitoring Committee
MRI: Magnetic Resonance Imaging
NCI: National Cancer Institute
OS: Overall Survival
ORR: Objective Response Rate
PD 1: programmed death 1
PDL 1: programmed death ligand 1
PFS: Progression-Free Survival
QoL: Quality of Life
RR: Response Rate
SPIRIT: Standard Protocol Items, Recommendations for Interventional Trials
VEGF: Vascular Epithelial Growth Factor

## Declarations

### Scientific approval

This trial was scientifically approved by ARCAGY-GINECO Intergroup (academic clinical research group specializing in gynecological oncology), accredited by INCa and supported by the Ligue contre le Cancer.
